# Accuracy of the registration of a digital dental cast or intraoral scan into a stereophotogrammetric image of the face: a systematic review

**DOI:** 10.1093/ejo/cjag057

**Published:** 2026-08-06

**Authors:** Anouk E H C Go, Rutger H Schepers, Hans L L Wellens, Johan Jansma, Anne-Marie Kuijpers-Jagtman

**Affiliations:** Department of Dentistry Orthodontics, University Medical Center Groningen, University of Groningen, Hanzeplein 1, 9713 GZ Groningen, The Netherlands; Department of Oral and Maxillofacial Surgery, Martini Hospital, Van Swietenplein 1, 9728 NT Groningen, The Netherlands; Department of Dentistry Orthodontics, University Medical Center Groningen, University of Groningen, Hanzeplein 1, 9713 GZ Groningen, The Netherlands; Department of Oral and Maxillofacial Surgery, Martini Hospital, Van Swietenplein 1, 9728 NT Groningen, The Netherlands; Department of Dentistry Orthodontics, University Medical Center Groningen, University of Groningen, Hanzeplein 1, 9713 GZ Groningen, The Netherlands; Department of Orthodontics and Dentofacial Orthopedics, School of Dental Medicine/Medical Faculty, University of Bern, Hochschulstrasse 4, 3012 Bern, Switzerland; Faculty of Dentistry, Universitas Indonesia, Campus Salemba, Jalan Salemba Raya No. 4, Jakarta 10430, Indonesia

**Keywords:** orthodontics, face, dental, imaging, three-dimensional, accuracy, trueness, precision, integration, CBCT, systematic review

## Abstract

**Background:**

Recent technological advances enable integration of digital dental casts or intraoral scans with 3D facial stereophotogrammetry. However, evidence on the accuracy of this integration remains limited.

**Objectives:**

To assess the level of evidence regarding the accuracy of the integration of a digital dental cast or intraoral scan into a 3D-facial image, in comparison to a full face CBCT scan in healthy growing and nongrowing patients.

**Search methods:**

MEDLINE (PubMed), EMBASE, Web of Science, Cochrane Library were searched. A manual search was performed of reference lists of included articles.

**Selection criteria:**

Inclusion criteria were studies investigating the integration of a digital dental cast or intraoral scan into a 3D stereophotogrammetric picture of the face, with a full face CBCT as a comparator. All study designs except case reports were included.

**Data collection and analysis:**

A pilot assessment was conducted, to synchronize the reviewers’ criteria for data extraction. After data extraction, a Risk of bias (RoB) assessment was performed using the Quality assessment of diagnostic accuracy studies (QUADAS-2) tool. Certainty of the evidence was assessed by the Grading of Recommendations, Assessment, Development and Evaluation (GRADE) tool.

**Results:**

Four eligible studies were identified and two more after updating the literature search. All reviewed studies confirmed feasibility of integrating intraoral scans with 3D facial images. The QUADAS-2 assessment revealed a high risk of bias for all included studies. Certainty of evidence concerning the accuracy was very low (GRADE). Due to heterogeneity concerning registration procedures and anatomical landmarks between the included studies, a qualitative description analysis of the results was made.

**Conclusions:**

The accuracy of the registration of a digital dental cast or intraoral scan into a facial scan is clinically acceptable. Most, but not all outcomes met the 2 mm or 2 degree threshold, depending on anatomical region, image acquisition, and measurement method. A 3D image of the face with an integrated digital dental cast or intraoral scan may be a promising radiation-free method for orthodontic diagnostics and treatment planning. However, due to the inconsistency in different registration methods and small sample sizes, the accuracy is yet to be further investigated.

**Clinical trials registration:**

INPLASY (INPLASY202420038)

## Introduction

Orthodontic treatment planning traditionally relies on a standard patient dataset that includes extraoral and intraoral photographs, a cephalogram, and an orthopantomogram (OPG) [1].

Recent years have seen significant technological innovation in 3D imaging, including the introduction of cone-beam computed tomography (CBCT), intra-oral scanning, and 3D facial scanning. Interestingly, despite these advancements, the core orthodontic diagnostic recommendations and requirements have remained consistent: both the American and European Boards of Orthodontists continue to require the above mentioned datasets as part of the clinical examinations for certification. A limitation of lateral cephalometric radiographs is that they offer a two-dimensional (2D) representation of inherently three-dimensional (3D) craniofacial structures. Moreover, they expose patients to ionizing radiation, which raises concerns under the ALARA principle (As Low as Reasonably Achievable), emphasizing the importance of reducing radiation exposure. Recent studies have questioned the routine necessity of cephalometric radiographs in orthodontic diagnostics, with some evidence suggesting that the cephalometric information may not significantly influence orthodontic treatment planning [2]. For example, Rischen *et al*. [3] demonstrated that cephalograms are not routinely needed for treatment planning in patients with Class II malocclusions. This has led to growing interest in exploring alternatives to cephalometric radiography, especially those that reduce radiation exposure.

One promising alternative is 3D stereophotogrammetry, a nonionizing imaging technique that produces accurate and reproducible 3D facial images [4, 5]. Additionally, intraoral digital dental scans have emerged as reliable substitutes for traditional plaster dental casts. Integrating digital dental casts or intraoral scans with 3D facial images has shown potential for enhancing orthodontic diagnostics without requiring X-ray imaging [6]. Furthermore, integration of intraoral scans, 3D facial images and CBCT scans has been found to be accurate [7]. However, CBCT imaging is not routinely utilized in orthodontics due to radiation concerns.

The accuracy of integrating a digital dental cast or an intraoral scan into a 3D facial image without the need of a CBCT scan, is crucial for its practical application. If proven accurate, this integrated 3D imaging approach could replace cephalograms in orthodontic diagnostics and treatment planning, enabling to create patient-specific treatment plans while significantly reducing the necessity for radiographic imaging.

This systematic review aims to evaluate the accuracy of integrating a digital dental cast or intraoral scan into a 3D-stereophotogrammetric facial image. Specifically, it seeks to determine whether this integration can serve as a viable alternative to cephalograms in orthodontic diagnostics.

## Materials and methods

### Protocol and registration

The study protocol was registered on the International Platform of Registered Systematic Review and Meta-analysis Protocols, INPLASY (https://inplasy.com), with registration number INPLASY202420038 [8]. The protocol was published 7 February 2024. The PRISMA 2020 guidelines were followed for the reporting of this systematic review [9, 10].

### Research question

The research question was formulated using the population, intervention, comparison, outcome (PICO) method and was as follows: ‘What is the accuracy (in degrees or millimeters) of the integration of a digital dental cast or intraoral scan into a 3D-facial image, in comparison to a full face CBCT scan in healthy growing and nongrowing patients (with any orthodontic malocclusion)?’

### Eligibility criteria

The inclusion criteria were the following:

- Population: Healthy growing and nongrowing human subjects of any age or sex and with any orthodontic malocclusion whereby the following records were present: a 3D-stereophotogrammetric facial image, a digital dental cast or intraoral scan and a CBCT.
- Intervention: The integration of a digital dental cast or intraoral scan into a 3D-facial image.
- Comparison: Full face CBCT scan including the area of the upper and lower dentition.
- Outcome: The deviation in millimeters or degrees of the three-dimensional position of the dentition from the digital dental cast or intraoral scan in the 3D facial image of the face in comparison to the CBCT's dentition, which served as the ‘gold standard’.

All study designs except case reports were included.

### Information sources and search strategy

A comprehensive search was conducted in MEDLINE (using PubMed), Embase, the Cochrane Library, and Web of Science. Gray literature was not searched. A hand-search of the references lists of the included studies was carried out. There were no restrictions on language or date of publication. The search was performed on 4 March 2024 and updated on 1 February 2025.

The search strategy was developed for MEDLINE and adjusted for the other databases (Supplementary Table S1).

### Study selection

Search results from the electronic databases were collected in EndNote X9 (2013, Clarivate Analytics, Philadelphia, USA). Duplicates were removed, following the de-duplication method designed by Bramer *et al*. [11]. The articles were then exported to Covidence (Covidence systematic review software, Veritas Health Innovation, Melbourne, Australia). Two reviewers (AG, AK) performed the study selection independently. A pilot assessment of ten studies was conducted jointly beforehand, to synchronize the reviewers’ criteria. The screening process was conducted in two steps: (i) title and abstract screening; (ii) Full-text screening of the remaining articles to determine if they met the inclusion criteria. Furthermore, the references of the selected studies were hand-searched to identify any additional relevant studies. Disagreements between the reviewers were resolved through discussion with a third reviewer (RS).

### Data extraction

A data extraction form was developed in Excel (Excel, Microsoft, Redmond, Washington, USA). The two authors (A.E.H.C.G. and A.M.K.-J.) independently extracted the following items: author, year of publication, country, sample size calculation, study group population, inclusion criteria, acquisition of dental model, acquisition of stereophotogrammetric pictures, registration technique, anatomical landmarks, results/outcome. Whenever information was incomplete or unavailable, the study's authors were contacted by email to obtain the necessary data. The effectiveness of the data extraction template was evaluated in advance in a data extraction pilot. In assessing the included articles, we applied the definitions of ISO 5725-1:2023 [12], whereby trueness refers to ‘the closeness of agreement between the expectation of test results and a true value’ and precision refers to the ‘closeness of agreement between independent test results obtained under stipulated conditions’.

### Risk of bias assessment of included studies

To assess the risk of bias (RoB) in the included studies, we used the Quality assessment of diagnostic accuracy studies—QUADAS-2 tool [13]. The QUADAS-2 was assessed on the patient selection, index test, reference standard and flow and timing. The RoB was assessed and classified as ‘low,’ ‘high’ or ‘unclear.’ Two authors (A.E.H.C.G. and R.H.S.) independently evaluated each study, and disagreements were solved by a third reviewer (A.M.K.-J.).

### Certainty of the evidence

The Grading of Recommendations, Assessment, Development, and Evaluation (GRADE) was used to judge the quality of the evidence [14], which was rated in five domains: study design, risk of bias, inconsistency, indirectness and imprecision. The classification per outcome was rated as high, moderate, low, or very low.

### Data synthesis

Heterogeneity was assessed based on registration procedures and the anatomical landmarks used. A descriptive analysis was carried out. A quantitative analysis of the findings was intended only when there is a substantial homogeneity in the included studies.

## Results

### Study search and selection

**Figure 1:**
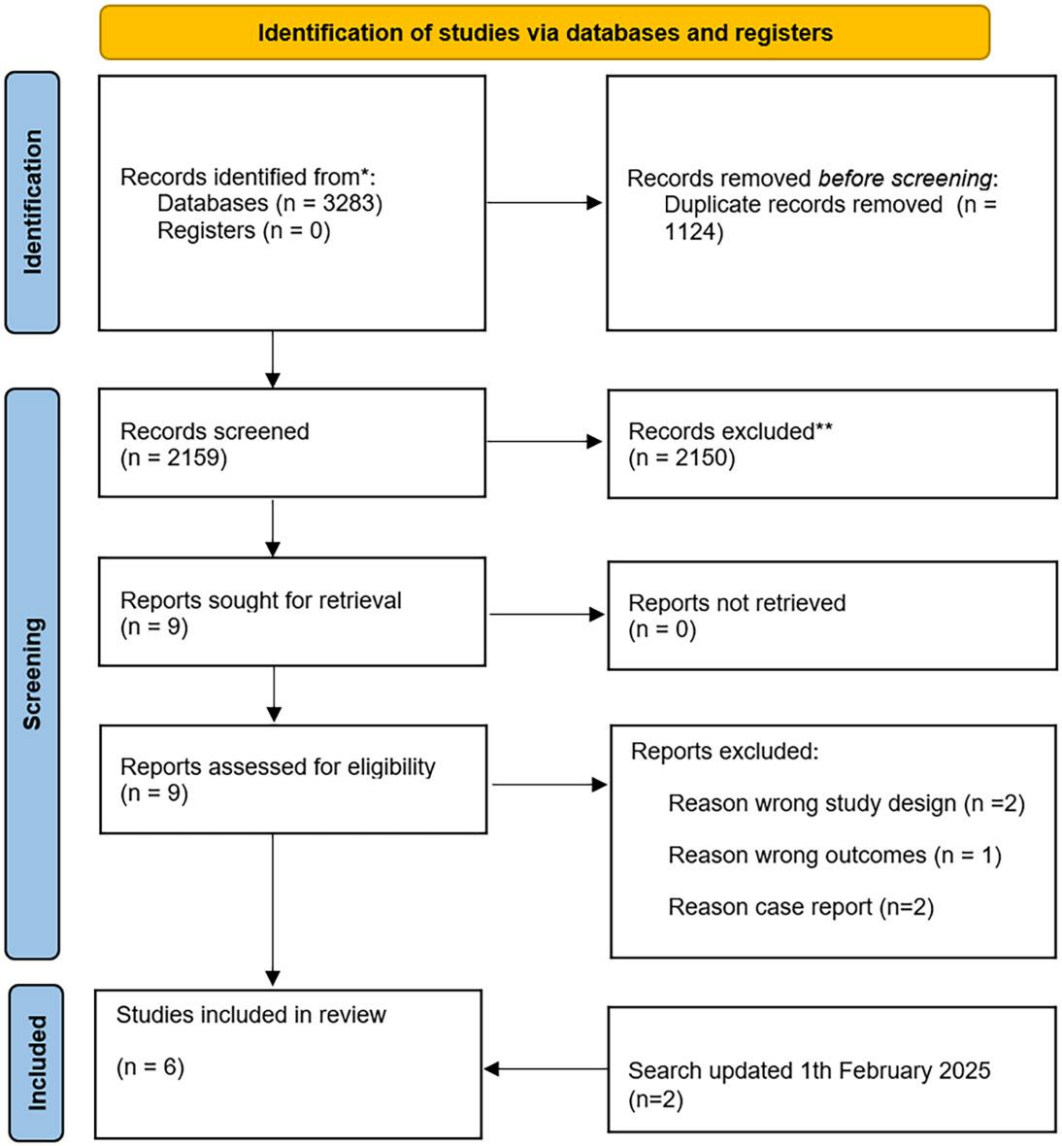
PRISMA flow diagram for the selection of studies.

The electronic search identified 3283 articles. The PRISMA flow diagram is presented in Fig. 1. After removing the duplicates, 2159 articles remained. After reading the title and abstract, 9 articles were selected for full text reading. All articles were retrieved. After full text reading four articles were included. In addition, the reference lists of all included studies were manually searched. The search was updated 1 February 2025. This update identified one additional eligible article. Screening of the reference list of the latter article yielded one further eligible study. So, a total of 6 articles were included (Table 1).

**Table 1:** Materials and methods of the included studies.

First author year Country	*N* Sex (F, M), age	Study design	Sample size calculation	Inclusion	Acquisition method dental model	Acquisition method 3D facial image	Registration procedures	Anatomical landmarks on CBCT and 3D facial image
Bechtold 2012 [15] Germany	*N* = 19 sex 13F, 6M age 13.1 ± 2.37 years	Prosp	No	Orthodontic patients with CBCT for diagnosis of tooth displacement or degenerative diseases of the TMJ, and photos and dental plaster casts.	Dental plaster cast digitized with model scanner (3D-Shape®, Erlangen, Germany)	2 images with 3D optical FaceSCAN3D system (3D-Shape, Erlangen, Germany) with transfer unit on bite fork (Amann Girrbach AG, Koblach, Austria) in rest	Ten step integration, using nasal tip and tragus points for superposition and refined by software Slim3D (3D-Shape®, Erlangen, Germany)	Measurements of 10 interlandmark distances. 4 facial landmarks: Inner eye corner (right and left) Subnasale Labrale superius 6 dental landmarks: Incisor (left and right) Canine (left and right) Molar (left and right)
Codari 2016 [16] Italy	*N* = 7 sex NR age NR	Retro	No	CBCT, stereophotogrammetric images, digitized dental plaster casts	Dental plaster cast digitized with laser scanner (Dental Wings series 3, Montreal Canada)	2 images with Vectra 3D, (Canfield Scientific, Fairfield, NJ, USA) lips open with cheek retractors in rest	ROI: upper anterior teeth in 3D-image and upper digital dental cast superimposed on at least 3 points using Procrustes method. Refined using the iterative closest point algorithm.	11 landmarks resulting in 14 distances between facial landmarks and landmarks on the upper dental arch and 14 on the lower dental arch. Landmarks: right and left Orbitale Pronasale Subnasale Sublabiale upper and lower inter-incisal distal vestibular cups of the first molars (16, 46, 26, 36)
Revilla-León 2022 [17] USA, Spain	*N* = 10 sex NR age ≥18 years	Prosp	No	Patients for mandibular dental implant, ≥ 18 years, English and Spanish speakers, dentate maxilla from first molar to first molar, only 1 posterior maxillary tooth missing, partially dentate in the mandible with stable occlusion, no contraindications for implant placement, no TMJ disease, no drug history affecting bone remodeling	Intra-oral scan with standardized scan body (TRIOS 4, 3Shape, Copenhagen, Denmark)	AFT Dental System (ScanBody Teeth and ScanBody Face, Sevilla, Spain) and Sat 3D system (Sat 3D; Sat 3D, Sevilla, Spain) reference image with standardized scan body on forehead and in the mouth (AFT ScanBodyFace) maximum intercuspation scan (closed mouth)	On 4 extraoral radiopaque markers on the face: glabella (Gb), Infraorbital canal left (LIO) and right (RIO) tip of the nose (TN) Iterative closest point technique between facial and intraoral digital scans	14 interlandmark distances measured in the CBCT scan and both 3D facial scan systems (AFT-system, Sat-3D system 6 facial measurements: Gb—TN Gb—IOR Gb—IOL TN—IOR TN—IOL IOR—IOL 8 dentofacial measurements IOR—16 IOR—13 TN—13 TN—11 TN—21 TN—23 IOL—23 IOR—26
Schobben 2025 [18] Netherlands	*N* = 18 sex 9F, 9 M age 16–59 years	Prosp	No	Patients for orthognathic surgery consultation	Intra oral scan (TRIOS 3, 3Shape, Copenhagen, Denmark)	3 stereophotogrammetric images (3dMD FaceSystem, 3dMD, London, UK at rest using cheek retractors with an impression model	Fusion of the models with the 3D software program 3DMedX® (v1.3.0.0, 3DLab Radboudumc, Nijmegen)	Differences between methods: subtracting the position of the maxilla in fusion model A (3dMD cheek retractors matched on 3dMD rest) minus the position in fusion model C (3dMD rest matched on CBCT); position in model B (3dMD impression model matched on 3dMD rest) minus position in model C; and position in model A minus position in model B
Xiao 2020 [19] China	*N* = 20 sex 14F, 6M age 24.5 ± 9.3 years	Prosp	No	CBCT for clinical reasons, complete permanent dentition, no history of orthodontic treatment	Dental plaster cast digitized with laser scanner (R900, 3Shape, Copenhagen, Denmark)	2 images with 3D optical FaceSCAN3D system (3D-Shape, Erlangen, Germany lips open with cheek retractors maximum intercuspation (closed mouth)	Registration of digital dental model into 3D facial image (cheek retractor) on upper anterior teeth; Registration of 3D facial image (at rest) onto 3D facial image (cheek retractor) using forehead, nasal root, zygoma regions	5 dental landmarks: mesio-incisal edge of 11, cusp tip of 13 and 23, mesio-buccal cusp of 16 and 26 in 3D coordinate system; the distances of x/y/z-axis between the dentofacial model and the CBCT was measured.
Ye H 2024 [20] China	*N* = 15 sex 9F, 6M mean age 25.5 years	Prosp	Yes	Participants with a complete maxillary dentition and without maxillofacial tumors or deformities	Intra oral scan (TRIOS 3, 3Shape, Copenhagen, Denmark)	3 images with 3D optical FaceSCAN3D system (3D-Shape, Erlangen, Germany in rest (closed mouth) lips open with cheek retractors in maximum intercuspation with a registered-block impression	on 5 radiopaque markers on the face: Glabella Zygion left and right Alare left and right Dental landmarks: 6 dental landmarks	25 linear distances were selected and measured by using a software program. 5 facial landmarks: Glabella Zygion (left and right) Alare (left and right) 6 dental landmarks: Incisor (left and right) Canine (left and right) Molar (left and right)

Abbreviations: 3D, 3-dimensional; CBCT, cone beam computed tomography; CI, confidence interval; F, Female; Gb, Glabella; IOL, Infraorbital canal left; IOR, Infraorbital canal right; M, Male; N, Number; NR, Not Reported, prosp, prospective; retro, retrospective; RMS, Root Mean Square; ROI, Region of Interest; TMJ, Temporomandibular Joint, TN, Tip of nose.

### Study characteristics

The subjects and methods of the included studies are summarized in Table 1. Of the six studies, published between 2012 and 2025, one study was retrospective [16], while the other five were prospective.

In all studies the primary inclusion criterion was the availability of a CBCT scan taken for various clinical reasons. Sample sizes ranged from *N* = 7 [16] to *N* = 20 [19]. The mean age of the participants was 18 years or older in three studies [17, 19, 20], while one study included participants aged 16–59 years [18]. In one study the age was 13.1 ± 2.37 years [15]. In one study the age range was not specified [16]. Four studies [15, 18–20] included both sexes, while two did not specify the participant's sex [16, 17].

Various methodologies were employed to investigate the superimposition process. Digital dental scans were obtained either through laser-scanned dental casts [15, 16, 19] or intra-oral scanning [17, 18, 20]. A 3D stereophotogrammetric image was acquired in all six studies, using different systems. Notably, Bechtold *et al*. [15], Xiao *et al*. [19] and Ye *et al*. [20] utilized the same FaceSCAN3D system (3D-Shape, Erlangen, Germany).

For stereophotogrammetric imaging, two studies used cheek retractors [16, 19], two made a reference picture with a polyvinyl siloxane impression model [15, 17], and two studies employed both methods [18, 20], although the impression model was made of wax. All six studies made a stereophotogrammetric picture at rest [15–20]. To superimpose the stereophotogrammetric picture at rest, two studies used radiopaque extraoral markers [17, 20], one study applied the Procrustes method as described in Hill *et al*. to an *unspecified* ‘Region of Interest’ [16], one study aligned models using the forehead, nasal root and zygoma regions [19], one study aligned models using the tip of the nose and the tragus points left and right [15] and one study used a software program 3DMedX® (v1.3.0.0, 3DLab Radboudumc, Nijmegen, The Netherlands) with the forehead used as a reference [18].

To assess the accuracy of the registration between the corresponding points on the stereophotogrammetric picture and the CBCT, five studies relied on anatomical landmarks, although each used different ones [15–17, 19, 20]. One study calculated intersurface distances from the registered models starting from the middle of the maxilla [18].

### Results of individual studies

Accuracy is a concept that includes trueness and precision. The results regarding the trueness and precision of the included studies are summarized in Table 2. All six reviewed studies confirm the feasibility of integrating intraoral scans with three-dimensional (3D) facial images [15–20]. As a result of the heterogeneity concerning registration procedures and the different anatomical landmarks between the included studies, a qualitative description analysis of the results was made. Deviations of <2 mm or 2 degrees were considered clinically acceptable [21].

**Table 2:** Results trueness and precision of the included studies.

First author Year	Trueness	Precision
Bechtold 2012 [15]	Mean interlandmark discrepancies scan vs CBCT: Vertical 0.07–0.78 mm Sagittal 0.09–1.46 mm	ICC for scan images: 0.996 ICC for CBCT 0.997
Codari 2016 [16]	Calculating 14 distances between landmarks on registered facial–dental scans and CBCT: Median distance 0.59 mm (IQR 0.43–0.73); Median 86.1% of points within 1 mm (81.6–92.1%)	NR
Revilla-León 2022 [17]	Range of 14 interlandmark discrepancies (mean ± SD) between CBCT and AFT or Sat-3D system: AFT minimum discrepancy 0.50 ± 0.05 mm; AFT maximum discrepancy 1.51 ± 0.14 mm Sat-3D: minimum discrepancy 0.59 ± 0.05 mm Sat-3D: maximum discrepancy 1.64 ± 0.09 mm	NR
Schobben 2025 [18]	Method A: cheek retractors Method B: model with tracer Method C: fusion model CBCT Method A and B compared with Method C Method A compared with C: Translational deviation: highest in Z-axis: 1.15 mm (95% CI 0.835 … 1.456) Method B compared with C: Translational deviation: highest in Y-axis: 1.85 mm (95% CI 1.224 … 2.467)	Method A compared with C (intra-observer): 1 out of 7 measurements sign diff: Z-axis: 0.254 mm (95%CI 0.032 … 0.477), *P* = 0.028 Method B compared with C (intra-observer): 5 out of 7 measurements sign diff Method A compared with C (interobserver) 0 out of 7 distances sign diff Method B compared with C (interobserver): 1 out of 7 measurements sign diff: pitch: 0.247^°^ (95% CI 0.007 … 0.486), *P* = 0.045)
Xiao 2020 [19]	CBCT is reference; compared with distances on dentofacial images RMS error: 0.37 mm; translational ≤0.42 mm; rotational: pitch 0.99°, yaw 0.25°, roll 0.53°	Intra-observer: ICC translation >0.998; rotation >0.905 Interobserver: ICC rotation >0.734
Ye H2024 [20]	Registered-block: 1.02 ± 1.24 mm (best); Anterior teeth: 2.35 ± 1.71 mm; CBCT reconstruction: 2.86 ± 1.61 mm	NR

Abbreviations: CBCT, cone beam computed tomography; ICC, Intraclass Correlation Coefficient; NR, not reported; RMS, root mean square; sign diff, significant difference.

The trueness or superimposition accuracy was assessed using various landmark-based measurements.

Bechtold *et al*. [15] repeated 3D measurements five times at 1-week intervals, using six dental and four facial landmarks. Mean distances between scan and CBCT measurements ranged vertically from 0.07 to 0.78 mm and sagittally from 0.09 to 1.46 mm. One distance (11 mesial–Inner eye corner right) had a marginal statistical significance (*P* = 0.041) but was considered not clinically relevant.

Codari *et al*. [16] calculated 14 distances between landmarks on registered facial–dental scans and CBCT. They reported a median distance of 0.59 mm with a median percentage of corresponding points of 86.1%, within 1 mm. Only the distance between the right Orbitale and tooth 36 was statistically significant.

Revilla-León *et al*. [17] documented trueness as measurements of 14 interlandmark discrepancies between a CBCT and the 3D facial images acquired with AFT and SAT-3D system, respectively. For the AFT system, the mean discrepancy was minimum 0.50 ± 0.05 mm and maximum 1.51 ± 0.14 mm. The Sat-3D system mean discrepancy was minimum 0.59 ± 0.05 mm and maximum 1.64 ± 0.09 mm. Their primary outcome was a comparison of two stereophotogrammetric systems, a technical rather than clinical endpoint, revealing comparable results between the systems.

Schobben *et al*. [18] assessed three methods: Method A using cheek retractors, Method B using a tracer and Method C was a fusion model with CBCT. Method A and Method B were compared with Method C. In the comparison between Method A and C, the translation deviation was the highest in the *Z*-axis: 1.15 mm (95% CI 0.835 … 1.456). In the comparison between Method B and C, the translation deviation was the highest in *Y*-axis: 1.85 mm (95% CI 1.224 … 2.467). Overall, 12 out of 14 measurements fell within the clinically acceptable thresholds of 2 mm or 2 degrees.

Xiao *et al*. [19] observed a mean translation error of ≤0.42 mm across all directions, with a mean rotational error of 0.99° pitch, 0.25° yaw and 0.53° roll. Errors were most pronounced in the molar region, Z-translation, and pitch rotation. Ye *et al*. [20] assessed the trueness of mean deviations for registered-block polyvinyl siloxane impressions (1.02 ± 1.24 mm), cheek retractors (2.35 ± 1.71 mm), and CBCT reconstructions (2.86 ± 1.61 mm). Notably, the registered-block method demonstrated the highest trueness, whereas CBCT reconstruction exhibited the lowest accuracy.

The precision was assessed in three studies [15, 18, 19]. Bechtold *et al*. [15] assessed the precision with the Intraclass correlation coefficient (ICC), which was reported 0.996 in scan images and 0.997 in CBCT. Schobben *et al*. [18] reported the precision with an intra-observer and interobserver analysis. In the analysis of the intra-observer reliability for method A compared with C one out of seven measurements was significantly different, for method B compared with C five out of seven were significantly different. The interobserver reliability analysis showed no significant difference for method A compared with method C and one out of seven in method B compared with C. In Xiao *et al*. [19] the intra-observer ICC was >0.998 for translation and >0.905 for rotation. The interobserver ICC was >0.734.

### Risk of bias in individual studies

The QUADAS-2 assessment for all included studies is summarized in Table 3. The complete description of the QUADAS-2 assessment is available upon request from the authors. In the domain patient selection, the study of Schobben *et al*. [18] had a low risk of bias. This can be explained by the consecutive inclusion of the patients. The studies of Revilla-León and Ye *et al*. [17, 20] classified as low on concerns about applicability. All other studies [15, 16, 19] were classified as high risk in this section.

**Table 3:** Quality assessment of diagnostic accuracy studies (QUADAS-2).

DOMAIN	Bechtold *et al*. 2012 [15]	Codari *et al*. 2016 [16]	Revilla-León *et al*. 2022 [17]	Schobben *et al*. 2025 [18]	Xiao et al. 2020 [19]	Ye et al. 2024 [20]
PATIENT SELECTION						
Risk of Bias	High	High	High	Low	High	High
Concerns about applicability	High	High	Low	High	High	Low
INDEX TEST						
Risk of Bias	High	Unclear	Unclear	Unclear	Unclear	Unclear
Concerns about applicability	High	High	High	High	High	High
REFERENCE STANDARD						
Risk of Bias	High	Low	Low	Low	Low	Low
Concerns about applicability	High	High	High	Low	High	High
FLOW AND TIMING						
Risk of Bias	High	High	High	High	High	High

For the domain ‘Index test’, all studies classified as a high or unclear risk of bias and were at high risk on concerns about applicability.

For the domain ‘reference standard’, Bechtold *et al*. [15] classified as a high risk of bias, while the other five articles had a low risk of bias [16–20]. The concerns about applicability were low in Schobben *et al*. [18], the other five were classified as high.

All included studies classified as high risk of bias on the domain ‘Flow and timing’. This can be explained due to missing data in all studies on the time interval between the inclusion, the acquisition of the 3D images and the reference CBCT scan.

### Level of evidence/level of certainty

#### Level of certainty

Accuracy, defined as a combination of both trueness and precision, was the primary outcome of interest in this review. Of the six included studies, only three reported both trueness and precision values and were therefore eligible for inclusion in the GRADE assessment for accuracy [15, 18, 19]. The overall certainty of evidence was rated as very low using the GRADE approach (Table 4). The evidence started as low (observational studies) and was downgraded due to serious risk of bias, as all studies had a high risk of bias according to the QUADAS-2 assessment. Additionally, we downgraded for indirectness, as some study populations and settings did not fully match with our research question. No downgrading for imprecision was applied, as the confidence intervals around effect estimates were sufficiently narrow and included clinically meaningful conclusions.

**Table 4:** GRADE evidence profile.

Outcome	No. of studies	Study design	Risk of bias	Inconsistency	Indirectness	Imprecision	No. of participants	Certainty
Accuracy	3 (Bechtold, Schobben, Xiao) [15, 18, 19]	Observational	Serious	Not serious	Serious	Not serious	57	●⚪⚪⚪ (Very low)

## Discussion

This systematic review aimed to evaluate the accuracy of integrating a digital dental cast or intraoral scan into a 3D stereophotogrammetric image of the face for orthodontic diagnostic purposes and treatment planning. The findings demonstrate that the accuracy is within acceptable clinical limits of 2 mm or 2 degrees deviation. In this systematic review we applied the commonly used thresholds of 2 mm and 2° to evaluate accuracy. While these cutoffs are widely accepted in cephalometric research and clinical practice, to our knowledge no primary validation study has formally established them. As also noted by van Bunningen *et al*. (2021) [21], the available literature consistently applies these thresholds without providing a methodological justification. We acknowledge that future research should aim to validate—and potentially refine—these criteria, particularly in light of ongoing technical improvements that may allow for stricter standards (e.g. 1 mm and 1°). Several methodological limitations were observed across the studies. The sample sizes were generally small (*n* = 7–20), and participant demographics were not consistently reported. Only three studies evaluated precision, showing good intra- and inter-observer reliability [15, 18, 19], though statistical significance in interobserver differences was still noted in several comparisons.

The reported trueness varied widely between studies, ranging from highly accurate measurements 0.07 mm [15] to deviations as large as 2.86 mm [20]. Although some outcomes fell within clinically acceptable thresholds of 2 mm or 2 degrees, the observed variation highlights the need for methodological standardization to enable meaningful comparison between studies. For example, Codari *et al*. [16], reported outcomes as percentages of corresponding points, which prevents direct comparison with studies using metric-based outcomes.

Standardizing is also needed for the definitions and use of the terms ‘trueness’ and ‘precision’. In this review the definitions from ISO 5725-1:2023 [12] were applied. However, definitions varied across the included studies. For example, Revilla-León *et al.* [17] defined trueness as ‘the average absolute discrepancy between the interlandmark distances measured on the CBCT and on the 3D patient representations’, which aligns with the ISO-definition. However, their definition of precision as ‘the interlandmark measurement discrepancies between the CBCT and the virtual 3D patient representations’ deviates from the ISO definition. The other five studies [15, 16, 18–20] did not explicitly define these terms, making comparisons between the included studies difficult and in some cases incompatible. Ye *et al.* [20] reported that measurements were performed three times, but these were combined averaged rather than analyzed separately for precision assessment. Therefore, we labeled the precision as ‘Not Reported’ (Table 2). Future studies should explicitly report the definitions of trueness and precision they apply, which would improve both readability and comparability across studies. All included studies confirmed the feasibility of superimposing intraoral scans into facial stereophotogrammetric images. Despite this promising potential, wide variations in the superimposition techniques and anatomical landmarks used across studies make it difficult to draw generalized conclusions. Superimpositions were achieved using different reference structures, including a region of interest [16], extra-oral markers [17, 20], anatomical regions [19], or 3D software algorithms [18]. This diversity of approaches has contributed to a high level of heterogeneity, precluding a quantitative synthesis of the results.

A pattern emerged suggesting that superimposition tends to be more accurate in the anterior region of the dentition than in the posterior region [16, 19]. However, inconsistencies in how translation, rotation, and angular deviations are reported complicate comparisons across studies. Notably, Revilla-León *et al*. [17] and Ye *et al*. [20] presented their findings within the context of technical system comparisons and did not address the clinical implications. In terms of practical relevance, the anterior region is generally easier to superimpose than the posterior molar region. A possible explanation is that the anterior teeth are more visible due to use of cheek retractors, which facilitates a more reliable best-fit alignment. In contrast, the more posterior, the pitch was more difficult to fit because the registration was fitted on a region which often falls outside the visible area captured in the images. Typically, cheek retractors expose the dentition only up to the first or second premolar. Furthermore, when using an impression model, a tilt along the *y*-axis may occur. This could be attributed to an occlusal cant introduced by the impression technique or by inaccuracies from the indirect registration.

Beyond the overall feasibility of integrating intraoral scans into 3D stereophotogrammetric images, the included studies consistently demonstrated that the acquisition method has a measurable influence on accuracy. Direct visualization of the anterior dentition, using cheek retractors, showed higher trueness in the anterior region, where dental surfaces are clearly exposed. However, cheek retractors introduce soft-tissue deformation and limit visibility beyond the premolar region, which negatively affected accuracy in the posterior areas and contributed to larger rotational deviations, particularly in pitch. Schobben *et al*. [18] quantified this effect, showing that the cheek-retractor method exceeded the clinically acceptable threshold in pitch rotation (2.51°) despite being acceptable in most other dimensions. Indirect methods reduce soft-tissue disturbance and improve precision, but introduce additional steps and potential registration chain errors. Furthermore, the overall certainty of evidence was rated as very low using the GRADE criteria. The main reasons for downgrading included serious to critical risk of bias, particularly regarding participant selection and lack of blinding during outcome assessment, as well as indirectness due to differences in study populations and clinical settings. While confidence intervals around reported measurements were typically narrow, the wide variability in techniques and outcome measures remains a significant concern.

Despite these limitations, the findings suggest that 3D stereophotogrammetry, when integrated with intraoral scans, may eventually offer a radiation-free alternative for specific diagnostic purposes in orthodontics. This approach aligns with the As Low as Diagnostically Acceptable, being Indication-oriented and Patient-specific (ALADAIP) principle [22]. It seeks to minimize radiation exposure whenever possible, especially given that a cephalogram is not necessarily routinely indicated for all orthodontic patients [2, 3]. The effective dose from a cephalogram is on average 2.0–10.0 μSv [23]. However, to put it in perspective, a conventional head CT scan is 860–1500 μSv. The average background radiation equivalent (D) is respectively <1 and 101–177 D. The dose of a cephalogram is low compared with the average background radiation, however it adds on to the cumulative doses. Previous studies have found that cumulative low-dose radiation exposure may be associated with triggering oxidative stress or potentially inducing damage to cellular DNA [24] or oral epithelium increasing the risk of carcinogenesis [25]. This is especially a risk in the mainly children dominated population of the orthodontist, with longer cumulative exposure over a lifetime and the higher radio-sensitivity.

However, several issues need to be addressed before clinical implementation can be recommended. These include the development of standardized protocols for image acquisition, for example with cheek retractors, and superimposition, identification of consistent anatomical landmarks, and validation of outcomes in larger, clinically relevant cohorts. Further research should also explore the potential of these integrated images to replace lateral cephalograms in treatment planning and outcome assessment.

### Limitations

The studies included in this review had several limitations. Ambiguous definitions of trueness and precision made it difficult to compare the included articles. The considerable methodological heterogeneity across studies prevented a meta-analysis. In addition, the overall sample size was small, limiting the generalizability of the findings. The methods used for 3D-image acquisition also varied, including direct acquisition with cheek retractors, in which the dentition was visible in the image, or indirect acquisition via a dental impression (the impression material is shown outside the mouth, reconstruction via the impression). Furthermore, the chosen anatomical landmarks differed across all studies.

### Future Recommendations

This review confirms the technical feasibility of integrating digital dental scans into stereophotogrammetric facial images, but methodological heterogeneity and low certainty of evidence hinder clinical application. Standardization of superimposition techniques and anatomical reference points is essential to enable comparison across studies. Future research should establish standardized protocols for image registration, reporting, and clinical validation to evaluate diagnostic and treatment planning outcomes using integrated 3D images. Addressing these gaps could advance a further minimizing of the radiation exposure for orthodontic patients and create a 3D integrated image which is patient-specific for diagnostics and treatment planning in orthodontics.

## Conclusion

The accuracy of the registration of a digital dental cast or intraoral scan into a facial scan is clinically acceptable. Most, but not all outcomes met the 2 mm or 2 degree threshold, depending on the anatomical region, image acquisition and measurement method. A 3D image of the face with an integrated digital dental cast or intraoral scan may be a promising radiation-free method for orthodontic diagnostics and treatment planning. However, due to the inconsistency in different registration methods and small sample sizes, the accuracy is yet to be further investigated.

## Supplementary Material

cjag057_Supplementary_Data

## Data Availability

The data that were generated for this study is reported in the article and in its online Supplementary material.

## Supplementary material

The supplementary material as mentioned in the publication is available on the website of the *European Journal of Orthodontics* online.
