# Supplementary material for: Accuracy of the registration of a digital dental cast or intraoral scan into a stereophotogrammetric image of the face: a systematic review
Source: Eur J Orthod. 2026 Aug 6;48(5):cjag057. doi: 10.1093/ejo/cjag057 (PMC13445612; doi:10.1093/ejo/cjag057)
Supplement: Supplementary file 1 [file cjag057_supplementary_data.docx]

Supplementary table 1. Database search strategy.

| Database | Search Strategy | Results |
| --- | --- | --- |
| PubMed | ("Face"[Mesh] OR Face[tiab] OR faces[tiab] OR facial*[tiab] OR dentofacial*[tiab] OR dental*[tiab] OR “intra-oral”[tiab]) AND ("Imaging, Three-Dimensional"[Mesh] OR tomogra*[tiab] OR 3d[tiab] OR “3-dimension*”[tiab] OR 3dmdface[tiab] OR “three-dimension*”[tiab] OR imag*[tiab] OR "Anthropometry"[Mesh] OR Anthropometr*[tiab] OR "Tomography, X-Ray Computed"[Mesh] OR photo*[tiab] OR scan*[tiab] OR stereophoto*[tiab] OR "Photogrammetry"[Mesh] OR "Image Processing, Computer-Assisted/methods"[Mesh]) AND ("Reproducibility of Results"[Mesh] OR accur*[tiab] OR valid* OR reproduc* OR "Feasibility Studies"[Mesh]) AND Integrat*[tiab] NOT ("animals"[MeSH]  NOT "humans"[MeSH]) NOT (invitro[ti] OR “in vitro”[ti] OR surger*[ti]) | 1029 reults |
| Embase | ('face'/exp OR (Face OR faces OR facial* OR dentofacial* OR dental* OR ‘intra-oral’):ab,ti,kw)  AND  ('three-dimensional imaging'/exp OR 'anthropometry'/exp OR 'x-ray computed tomography'/exp OR 'photogrammetry'/exp OR 'image processing'/exp OR (tomogra* OR 3d OR ‘3-dimension*’ OR 3dmdface OR ‘three-dimension*’ OR imag* OR Anthropometr* OR photo* OR scan* OR stereophoto*):ab,ti,kw)  AND  ('reproducibility'/exp OR 'feasibility study'/exp OR (accur* OR valid* OR reproduc*):ab,ti,kw)  AND  Integrat*:ab,ti,kw NOT ('animal'/exp NOT 'human'/exp) NOT (invitro:ti OR ‘in vitro’:ti OR surger*:ti) NOT 'conference abstract'/it | 989 results |
| Web of Science | TS=(Face OR faces OR facial* OR dentofacial* OR dental* OR “intra-oral”)  AND  TS=(tomogra* OR “3d” OR “3-dimension*” OR “3dmdface” OR “three-dimension*” OR “imag*” OR “Anthropometr*” OR “photo*” OR scan* OR “stereophoto*”)  AND  TS=(“valid*” OR “reproduc*” OR “Feasibility stud*”)  AND  TS=Integrat*  NOT TI=(“invitro” OR “in vitro” OR “surger*”) | 1191 results |
| Cochrane | (Face OR faces OR facial* OR dentofacial* OR dental* OR “intra-oral”)  AND  (tomogra* OR “3d” OR “3-dimension*” OR “3-dimensions” OR 3dmdface OR “three-dimension” OR “three-dimensions” OR imag* OR Anthropometr* OR photo* OR scan* OR stereophoto*)  AND  (“valid*” OR “reproduc*” OR “Feasibility study” OR “Feasibility studies”)  AND  Integrat* | 74 results |
